# Comparison of subfoveal choroidal thickness in eyes with CRVO and BRVO

**DOI:** 10.1186/s12886-019-1143-9

**Published:** 2019-06-21

**Authors:** Fen Tang, Fan Xu, Haibin Zhong, Xin Zhao, Mingliang Lv, Ke Yang, Chaolan Shen, Hui Huang, Jian Lv, Siming Zeng, Min Li, Qi Chen

**Affiliations:** 1 Department of Ophthalmology, People’s Hospital of Guangxi Zhuang Autonomous Region, Nanning, Guangxi China; 2 Department of Ophthalmology, Renmin Hospital of Wuhan University, Wuhan, Hubei China

**Keywords:** Subfoveal choroidal thickness, Retinal vein occlusion, Macular edema, Ranibizumab

## Abstract

**Background:**

To evaluate the subfoveal choroidal thickness (SFCT) in eyes with macular edema (ME) secondary to retinal vein occlusion(RVO), and to investigate the short term response after a single intravitreal ranibizumab (IVR) injection. What is more, to compare SFCT and SFCT change between central RVO (CRVO) and branch RVO (BRVO).

**Methods:**

In the retrospective study, we had collected 36-six treatment-naïve patients with unilateral ME secondary to RVO (including 19 CRVO and 17 BRVO). All patients had received IVR injection after newly diagnosed. The SFCT was measured at the onset and after 2 weeks of IVR injection. Paired *t* test was performed to compare the SFCT of RVO eyes and fellow eyes, as well as the SFCT of pre-injection and post-injection. In further, independent *t* test was used to compare SFCT and SFCT change between CRVO eyes and BRVO eyes.

**Results:**

The mean SFCT at the onset was 326.03 ± 30.86 μm in CRVO eyes, which was significantly thicker than that in contralateral fellow eyes (*p* < 0.01, paired *t* test), and reduced to 294.15 ± 30.83 μm rapidly after 2 weeks of IVR injection (*p* < 0.01, paired *t* test). Similarly, the SFCT in BRVO eyes was significantly thicker than that in contralateral eyes at the onset, and decreased significantly after IVR injection. However, our findings showed that there was no statistically significant difference in SFCT and SFCT reduction after IVR injection between CRVO eyes and BRVO eyes.

**Conclusions:**

The SFCT in eyes with ME secondary to CRVO and BRVO was significantly thicker than that in fellow eyes, and decreased significantly within a short time in response to a single IVR injection. In further, the study showed that SFCT and SFCT change had no correlation with RVO subtypes.

## Background

Retinal vein occlusion (RVO) is a retinal vascular disorder characterized by obstruction of the retinal venous system, often associated with hypertension and coagulation abnormalities [1, 2]. It is a common cause of visual handicap in the elderly throughout the world [3], and could be subdivided into central RVO (CRVO), branch RVO (BRVO) and hemi RVO (HRVO) according to the location of blockage [4]. Moreover, both CRVO and BRVO can be further classified into non-ischemic subtype and ischemic subtype based on the amount of retinal capillary perfusion [5]. Macular edema (ME) is one of the prominent complication in patients with ischemic RVO and can cause severe impairment of central vision [6]. Various treatment modalities had been used to treat ME, anti-vascular endothelial growth factor (VEGF) therapy had been demonstrated to be safe and effective among these available therapies [7–10].

The eyes with RVO may have abnormal choroidal vasculature, due to hydrostatic pressure and VEGF level [11]. Several studies had investigated subfoveal choroidal thickness (SFCT) in CRVO eyes and BRVO eyes, however, the results were contradictory. Some studies found that SFCT of affected RVO eyes had no significant difference compared with that of unaffected fellow eyes [11]. However, other studies showed that SFCT of RVO eyes was significantly thicker than that of unaffected fellow eyes [12] [13]. Besides, SFCT change after anti-VEGF therapy was also contradictory [14, 15]. Most of the studies reported that the SFCT was decreased significantly after anti-VEGF treatment [12, 16], while a few studies reported that SFCT didn’t decrease after anti-VEGF treatment [9]. Thus, these contradictory results warrant further investigation.

VEGF level was demonstrated as the principal factor which contribute to SFCT change [11]. Elevated VEGF expression could lead to increased capillary permeability and leakage in retina and choroid [2, 17], is critically involved in the pathogenesis of ME secondary to RVO [18, 19]. Franco-Cardenas and colleagues found that ischemic index in CRVO was much higher than that in BRVO [20], what is more, Yasuda and colleagues found that aqueous VEGF concentration in CRVO eye was significantly higher than that in BRVO eye [21], these studies suggested that retinal ischemia in CRVO was more severe than that in BRVO. Therefore, we assume that SFCT of CRVO eye may be thicker than that of BRVO eye, however, it is uncertain and need to be demonstrated.

The present study was aimed to further investigate the SFCT in CRVO and BRVO eyes respectively, and to evaluate its short term response after a single IVR injection. More importantly, to compare SFCT and SFCT change after IVR injection between CRVO eye and BRVO eye.

## Methods

In the retrospective case series, we collected and evaluated the data of 36 patients with unilateral ME secondary to RVO. Nineteen patients had CRVO, and 17 patients had BRVO. The diagnosis was determined according to the fundus examination and fluorescein angiography. Inclusion criteria were as the follows: (1) the age ranged from 50 to 70 years; (2) recent-onset (less than 1.5 months) and treatment-naïve when presented to the hospital; (3) was ischemic subtype and had received at least one intravitreal ranibizumab injection after newly diagnosed; (4) had follow-up of at least 2 weeks; (5) had comprehensive ophthalmic examinations before and after treatment. Patients were excluded if their fellow eyes had any macular disorder such as age-related degeneration (AMD), polypoidal choroidal vasculopathy (PCV) or central serous chorioretinopathy (CSC). Patients were also excluded if the affected eyes or fellow eyes had any of the following criteria: (1) axial length > 26.00 mm or < 22.00 mm; (2) a history of pars plana vitrectomy or other intraocular surgeries within half year. The present study followed the tenets of the declaration of Helsinki and was approved by the ethics committee in hospital. The subjects had been informed written consent on the study.

Data collected from patients’ medical records included age, axial length, gender, systemic diseases, and SFCT value at baseline and after 2 weeks of IVR injection. All patients had undergone standardized ophthalmic examinations, including best-corrected visual acuity (BCVA), intraocular pressure (IOP), slit-lamp biomicroscopy, funduscopic, fluorescein angiography (Heidelberg retina angiograph; Heidelberg Engineering Inc., Dossenheim, Germany), and enhanced depth imaging optical coherence tomography (EDI-OCT) (Heidelberg Engineering Inc., Dossenheim, Germany). BCVA was measured by the Early Treatment Diabetic Retinopathy Study (EDTRS). They had received intravitreal ranibizumab injection (Lucentis, 0.05 ml, 0.5 mg) after newly diagnosed. After that, the following treatment strategies were varied based on clinically relevant benefits and risks, patients’ anticipated visiting compliance, and the factor that whether the patients could afford the cost of ranibizumab. During the follow -up period, some of the patients were administered IVR injection monthly for three times, others received corticosteroids injection or laser photocoagulation. Nine patients with CRVO had received continuous IVR injection monthly for three times, whereas six patients with BRVO had received this treatment regimen. There were eight patients in both CRVO and BRVO groups who were administered corticosteroids injection or laser photocoagulation due to cost issue. Besides, five patients (including two CRVO and three BRVO) had lost to follow-up.

The demographic characteristics and SFCT value were collected. SFCT was measured from the outer border of the pigment epithelium to the choroidal scleral boundary, it was measured by 2 observers independently, and was recorded with the mean value. Statistical analysis was performed using Statistical Package for Social Science (SPSS) software (version 20.0, SPSS, Inc., Chicago, IL, USA). Continuous variables of the demographic characteristics were displayed as mean ± standard deviation (SD), categorical variables were displayed as the number of subjects and its percentage. Difference between continuous variables was analyzed by independent *t* test, and Chi-square test was used for categorical variables. The SFCT values were displayed as mean ± standard deviation (SD). The paired *t*-test was used to determine the difference in SFCT between RVO eye and its fellow eye. The SFCT between pre-injection and post-injection was also compared by paired *t*-test. The SFCT and SFCT change were compared between CRVO eye and BRVO eye by independent *t*-test. *P* value < 0.05 was considered statistically significant.

## Results

Choroidal thickness was associated with the demographic characteristics of subjects. Axial length is an important predictor factor for the macular choroidal thickness, previous studies had demonstrated that the eyes with longer axial length would have thinner choroidal thickness [22, 23]. Age is another important factor, previous studies reported that choroidal thickness would decrease 10-15 μm [24] or 20- 26 μm [25, 26] with age getting each 10 years older. As is summarized in Table 1, no statistically difference was founded in axial length and age between CRVO group and BRVO group (*p* < 0.05, independent *t* test), suggesting that the two groups were well balanced on axial length and age. Besides, there were no difference in gender distribution and smoking percentage between the two groups (*p* < 0.05, Chi-square test). The percentage of systemic diseases (such as hypertension, diabetes, and abnormal coagulation) had no statistically difference between the two groups. Additionally, the BCVA and IOP at baseline were shown in Table 1, no significant difference was found between CRVO and BRVO group.

**Table 1:** The patients’ demographic characteristics

	CRVO (*n* = 19)	BRVO (*n* = 17)	P
Age, years (Mean ± SD)	57.37 ± 9.75	56.53 ± 8.04	NS^a^
Gender, Male (%)	12(63.16%)	10(58.82%)	NS^b^
Axial length, mm (Mean ± SD)	23.55 ± 1.06	23.92 ± 1.03	NS^a^
Ever smoker (n, %)	6(31.58%)	8(47.06%)	NS^b^
Systemic diseases			NS^b^
Hypertension (n, %)	11(57.89%)	8(47.06%)	NS^b^
Diabetes (n, %)	3(15.79%)	4(23.53%)	NS^b^
Abnormal coagulation (n, %)	4(21.05%)	3(17.65%)	NS^b^
BCVA(EDTRS letters)	48.2 ± 19.4	50.2 ± 11.3	NS^a^
IOP(mmHg)	14.2 ± 2.5	15.4 ± 3.0	NS^a^

^a^ Independent *t* test, ^b^ Chi-square test

**Fig. 1:**
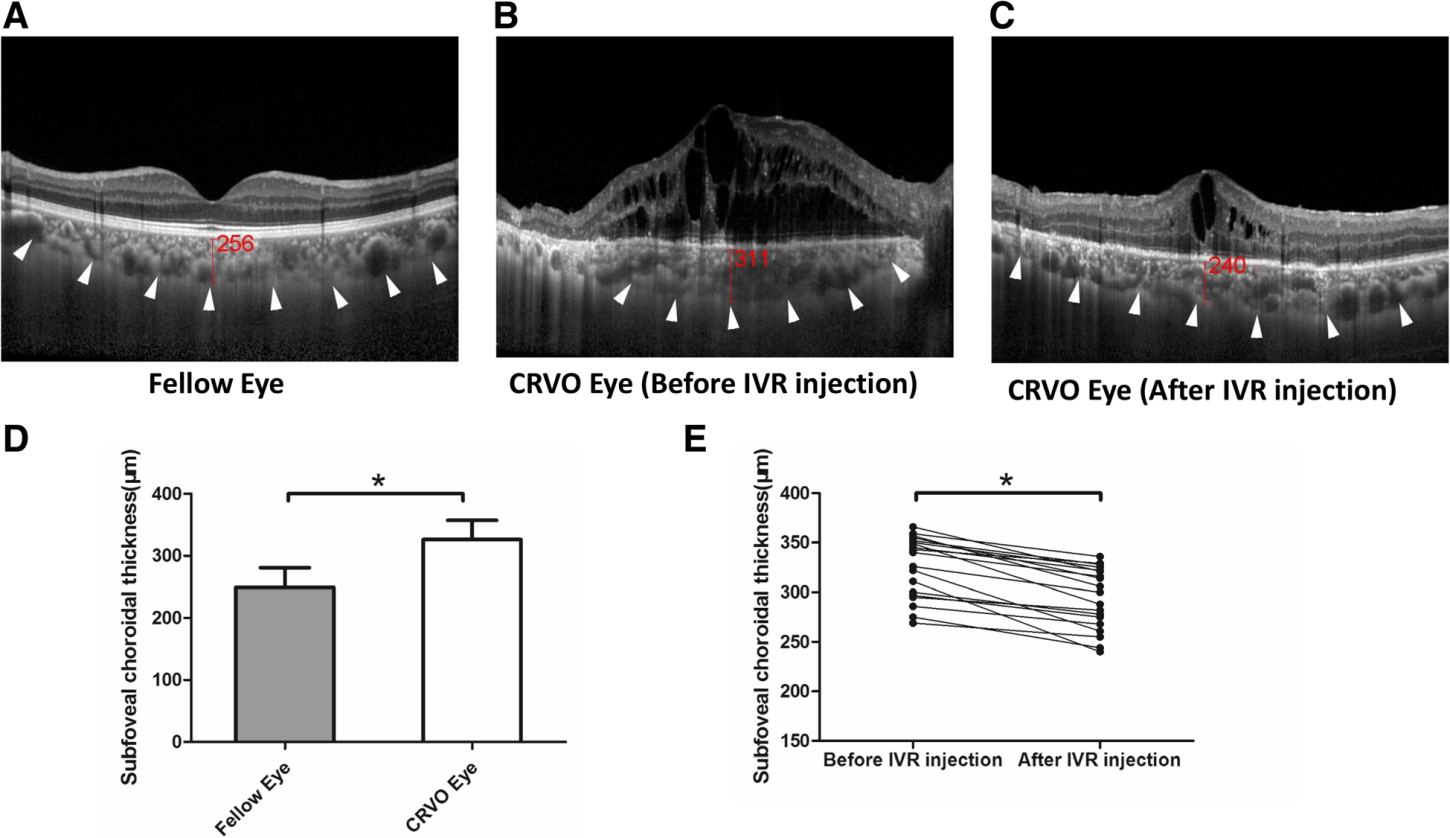
Subfoveal choroidal thickness (SFCT) in patients with macular edema secondary to CRVO. **a** The representative EDI-OCT image of unaffected contralateral eye; **b** The representative EDI-OCT image of affected CRVO eye before IVR injection; **c** The representative EDI-OCT image of affected CRVO eye after IVR injection; White arrowheads point the choroid-scleral junction. SFCT was determined as the vertical distance from the hyperreflective line of the retinal pigment epithelium to the line of the choroid-scleral junction centered on the fovea, it was measured by 2 observers independently, and was recorded with the mean value. **d** Comparison of SFCT between the unaffected fellow eyes and CRVO eyes. **e** Comparison of SFCT in CRVO eyes between pre-IVR injection and post-IVR injection. The values were displayed as mean ± standard deviation (SD). The paired *t* test was used to evaluate the differences. *p < 0.05 was considered statistically significant

Representative EDI-OCT images of CRVO are shown in Fig. 1a, b and c. Compared with unaffected fellow eyes, the SFCT of CRVO eyes was significantly thicker than that of fellow eyes, the mean SFCT of CRVO and fellow eyes were 326.03 ± 30.86 μm and 249.29 ± 31.55 μm, respectively (*p* < 0.001, paired *t* test). However, after 2 weeks of IVR injection, the mean SFCT of CRVO eyes reduced to 294.15 ± 30.83 μm, which was significantly thinner than that before treatment (Fig. 1d, *p* < 0.001, paired *t* test).

**Fig. 2:**
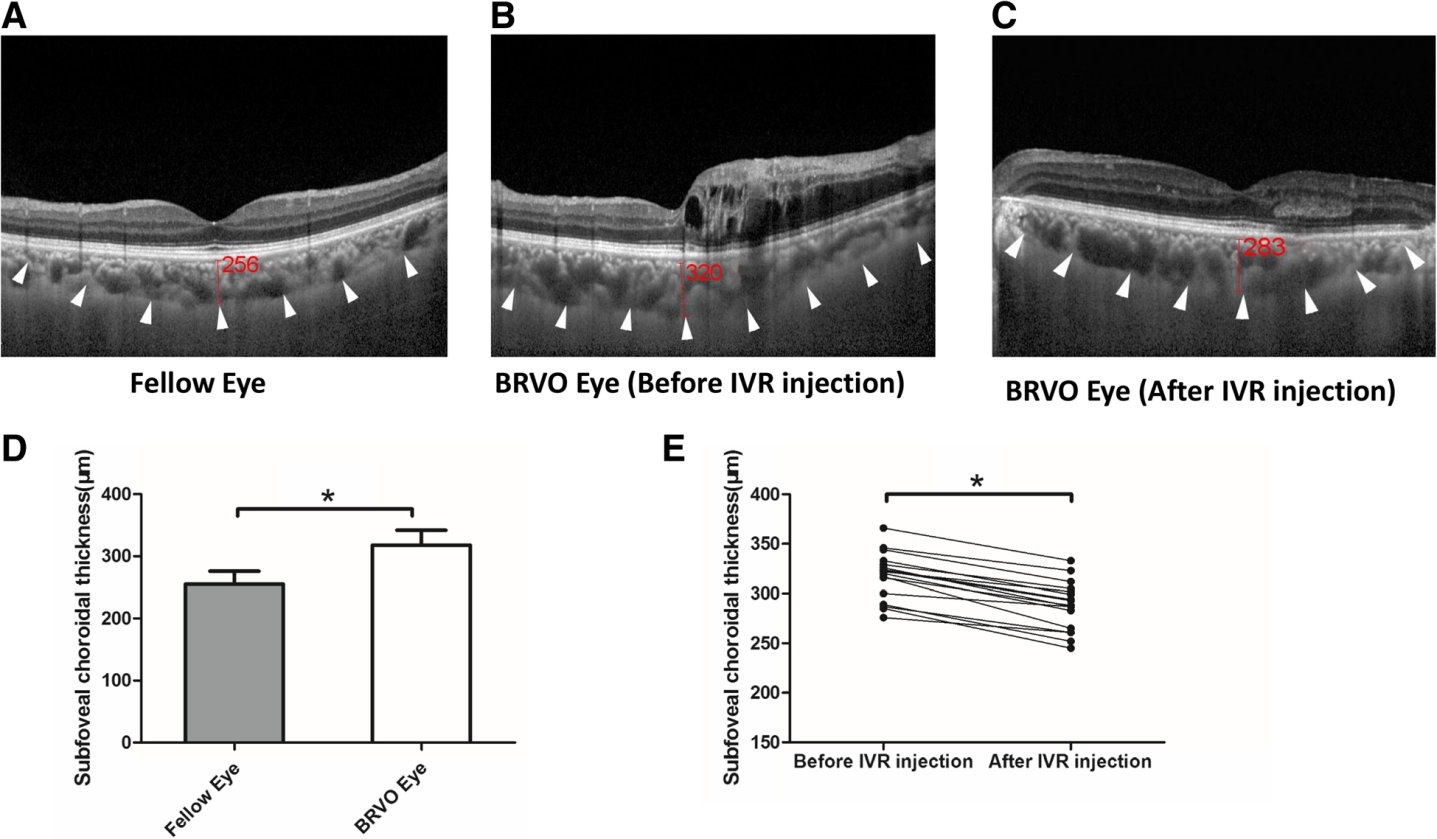
Subfoveal choroidal thickness (SFCT) in patients with macular edema secondary to BRVO. **a** The representative EDI-OCT image of unaffected contralateral eye; **b** The representative EDI-OCT image of affected BRVO eye before IVR injection; **c** The representative EDI-OCT image of affected BRVO eye after IVR injection; White arrowheads point the choroid-scleral junction. SFCT was determined as the vertical distance from the hyperreflective line of the retinal pigment epithelium to the line of the choroid-scleral junction centered on the fovea, it was measured by 2 observers independently, and was recorded with the mean value. **d** Comparison of SFCT between the unaffected fellow eyes and BRVO eyes. **d** Comparison of SFCT in BRVO eyes between pre-IVR injection and post-IVR injection. The values were displayed as mean ± standard deviation (SD). The paired *t* test was used to evaluate the differences. *p < 0.05 was considered statistically significant

Similarly, the SFCT of BRVO eyes was significantly thicker than that of fellow eyes, the mean SFCT of BRVO eyes and fellow eyes were 317.78 ± 24.09 μm and 255.21 ± 20.40 μm, respectively (Fig. 2d, *p* < 0.001, paired *t* test). Moreover, the SFCT of BRVO eyes reduced to 287.65 ± 24.42 μm rapidly after IVR injection (Fig. 2e, *p* < 0.001, paired *t* test).

**Fig. 3:**
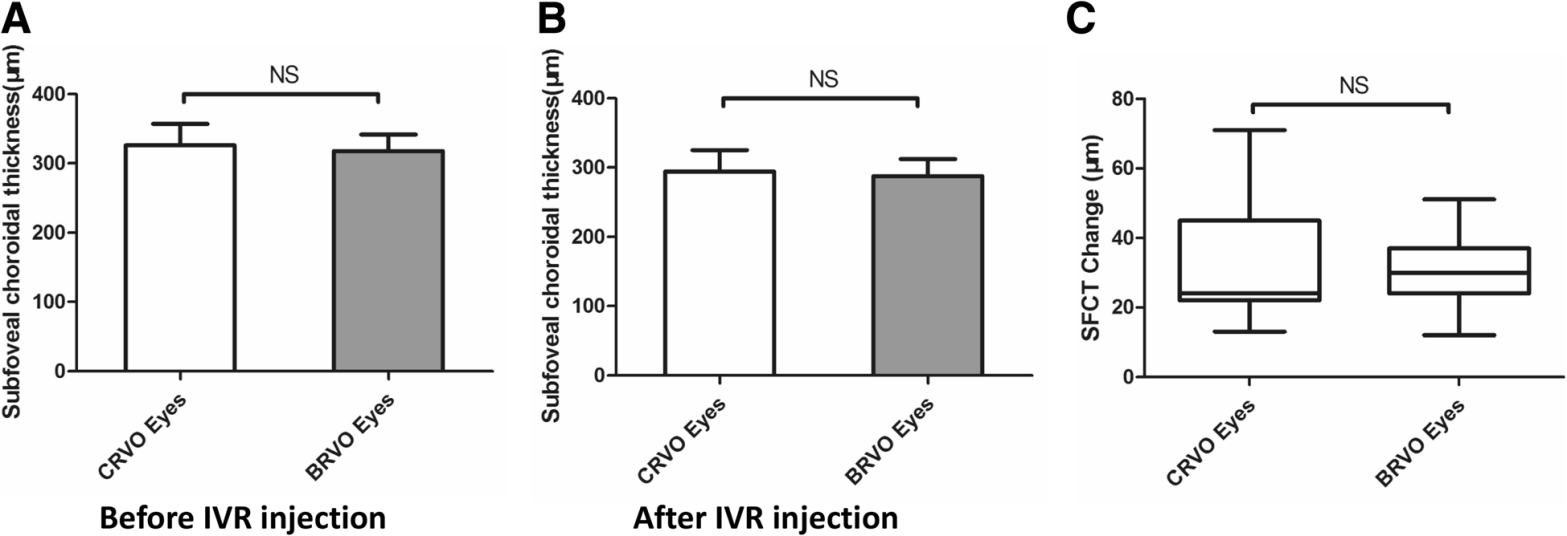
There was no significant difference in SFCT and SFCT change between CRVO group and BRVO group. **a** Comparison of SFCT before IVR injection between CRVO group and BRVO group; **b** Comparison of SFCT after IVR injection between CRVO group and BRVO group; **c** Comparison of SFCT change between CRVO group and BRVO group. The independent *t* test was used to evaluate the difference. *p* > 0.05 was considered no significant difference (NS)

As was shown in Fig. 1 and Fig. 2, the SFCT in CRVO eyes and BRVO eyes showed a similar change trend, the thicker SFCT restored after IVR injection. In further, we compared SFCT and SFCT change between CRVO eyes and BRVO eyes. Unexpectedly, although the SFCT of CRVO eyes (326.03 μm) was slightly thicker than that of BRVO eyes (317.78 μm) at the onset, no significant difference was found between them (Fig. 3 a, *p* > 0.05, independent *t* test). The SFCT reduction after treatment were 31.88 μm in CRVO eyes and 30.13 μm in BRVO eyes, respectively. There was also no statistically significant difference in SFCT reduction between these two groups. (Fig. 3 b, C, p > 0.05, independent *t* test).

## Discussion

The present study showed that the SFCT in eyes with ME secondary to RVO (including CRVO and BRVO) was significantly thicker than that in unaffected fellow eyes, and decreased rapidly within a short term in response to a single IVR injection, indicating that subfoveal choroid may be involved in the progress of ME secondary to RVO. The SFCT reduction after IVR was mainly caused by ranibizumab, which could permeate the retinal layer and extend to the choroid [27]. Furthermore, the SFCT and SFCT change were compared between CRVO group and BRVO group, no statistically significant difference was found, indicating that SFCT didn’t have correlation with RVO subtype.

Macular choroidal thickness was correlated with disease severity and prognosis, EDI-OCT could provide a noninvasive method to evaluate the choroidal thickness in vivo [28, 29]. Over the past several years, many studies had investigated the SFCT in macular-involved diseases. It was reported that the eyes with idiopathic macular hole [30] and dry AMD [31] had reduced SFCT, whereas the eyes with central serous chorioretinopathy (CSC) [32] and Vogt- Koyanagi-Harada (VKH) [33] had increased SFCT. ME is mainly caused by diabetic retinopathy and RVO. Previous studies showed that the SFCT in diabetic macular edema was thinner than that in normal eye [34], and was significantly correlated with the disease severity [35, 36]. With respect to ME secondary to RVO, there had been several studies to investigate choroidal thickness and the role of choroid in RVO eyes. As was mentioned above, Tsuiki and Coban Karatas found that the macular choroidal thickness in RVO eyes was thicker than that in unaffected fellow eyes [12, 13, 28]. In contrast, Du KF and colleagues reported that no significant difference was found between RVO eyes and its fellow eyes [11]. One of the explanation for the conflicting result is the difference RVO phase, the subjects recruited by Tsuiki and Coban Karatas were at acute phase, while the study conducted by Du KF included the patients at longstanding and acute phase, the discrepancy between these studies may be contributed to the patients at longstanding phase. In our study, we collected the resent-onset and treatment-naïve patients, who were at acute phase, and our results were consistent with Tsuiki’s findings. Furthermore, several studies demonstrated that choroidal thickness in RVO eyes decreased significantly following anti-VEGF treatment [12, 13, 16, 37], however, Park Jongyeop and colleagues reported that no SFCT change was found after anti-VEGF treatment [9]. The possible cause of this conflicting result might be the different follow-up period. Park Jongyeop evaluated SFCT after 12 months of treatment, while other studies evaluated it within a short follow-up period (ranged 1 month to 6 months). Our study evaluated SFCT after 2 weeks of IVR injection, which was a much shorter follow-up period. Our study still yield the similar finding with the studies which evaluated SFCT in the short term. The hypothesis is that SFCT may decrease in the short term after anti-VEGF treatment, and may restore in the long term. However, further investigation is needed to demonstrate it.

The initial choroidal thickness can be served as a biomarker of disease severity and a predictor of prognosis [35, 36, 38, 39]. Although there were several studies to evaluate the choroidal thickness in RVO, they focused on CRVO or BRVO separately [40, 41]. It had been demonstrated that CRVO eyes had higher ischemic index and VEGF level compared with BRVO eyes. Moreover, increased VEGF would induce vascular hyperpermeability and dilated vessel in choroid layer, which is the main cause of increased choroidal thickness [42, 43]. Thus, it is supposed that the higher the VEGF level is, the thicker the choroidal thickness become. Therefore, we speculated that the SFCT of CRVO eyes might be thicker than that of BRVO eyes. However, our findings didn’t show statistically significant difference between CRVO and BRVO. The possible reasons may be as follows:(1) The patients we collected were ischemic subtype, VEGF level in both CRVO and BRVO eyes was very high; (2) The sample size in each group was too small to detect a significant difference; (3) Besides VEGF, other unknown factors might contribute to choroidal thickness change. Overall, the exact relationship between choroidal thickness and RVO severity require further investigation in the future study.

The present study had several limitations. First, the small sample size, short follow-up period and retrospectively designed study are the drawbacks. The prospective study with large number of subjects and long term follow-up is required in the future. Second, we only collected the patients with ME secondary to ischemic RVO, the patients included are not representative of the population of CRVO and BRVO, they only represent a small population of RVO patients who have the ME complication, this could be a selection bias. In order to further determine the exact relationship between SFCT and disease severity, the patients with non-ischemic RVO, the patients without ME and the patients with other complications (such as neovascularization and glaucoma) should be included in the future study. Third, choroidal thickness can present diurnal variation, the SFCT value is correlated with the measurement time, however, the EDI-OCT for all subjects was not performed within the same range in the study, this could be a possible bias. Fourth, a few subjects with diabetes had been included, although they did not have any sign of diabetic retinopathy according to comprehensive ophthalmic examinations, a potential bias might occur.

## Conclusion

In conclusion, in recent-onset and treatment- naïve patients with ME secondary to RVO, the SFCT in affected eyes was statistically thicker than that in its unaffected contralateral eyes, and restored rapidly after 2 weeks of a single IVR injection. Our study may help to elucidate the conflicting results about the SFCT and SFCT change after anti-VEGF therapy. In further, our findings showed that there was no significant difference in SFCT and SFCT reduction between CRVO eyes and BRVO eyes, further study is still needed to investigate the exact relationship between SFCT and RVO severity.

## Data Availability

The datasets used and analyzed during the current study are available from the corresponding author on reasonable request.

## Abbreviations

AMD: Age-Related Degeneration
BRVO: Branch Retinal Vein Occlusion
CRVO: Central Retinal Vein Occlusion
CSC: Central Serous Chorioretinopathy
EDI-OCT: Enhanced Depth Imaging Optical Coherence Tomography
IVR: Intravitreal Ranibizumab
PCV: Polypoidal Choroidal Vasculopathy
RVO: Retinal Vein Occlusion
SFCT: Subfoveal Choroidal Thickness
VEGF: Vascular Endothelial Growth Factor
